# Chaetocin Promotes Osteogenic Differentiation via Modulating Wnt/Beta-Catenin Signaling in Mesenchymal Stem Cells

**DOI:** 10.1155/2021/8888416

**Published:** 2021-02-06

**Authors:** Youde Liang, Xin Liu, Ruiping Zhou, Dawei Song, Yi-Zhou Jiang, Weiwei Xue

**Affiliations:** ^1^Department of Stomatology, The Seventh People's Hospital of Shenzhen, China; ^2^Institute for Advanced Study, Shenzhen University, Shenzhen, China

## Abstract

Mesenchymal stemXin cells (MSCs) are a great cell source for bone regeneration. Although combining MSCs with growth factors and scaffolds provides a useful clinical strategy for bone tissue engineering, the efficiency of MSC osteogenic differentiation remains to be improved. Epigenetic modification is related to the differentiation ability of MSCs during osteogenic induction. In this study, we evaluate the effect of Chaetocin, an inhibitor of lysine-specific histone methyltransferases, on the differentiation of MSCs. We found that MSCs treated with Chaetocin demonstrated increased osteogenic ability and reduced adipogenic ability. The expression of osteogenic markers (Runx2 and OPN) was induced in MSCs by Chaetocin during osteogenic induction. Moveover, treatment of Chaetocin in MSCs improves Wnt/*β*-catenin signaling pathways and its downstream targets. Finally, we showed increased bone formation of MSC and Wnt/*β*-catenin signaling activity by treatment of Chaetocin using in vivo bone formation assays. Our data uncovered a critical role of Chaetocin in MSC osteogenic differentiation and provide new insights into bone tissue regeneration and repair.

## 1. Introduction

Bone healing is a complicated process and not always completely satisfactory. Repair of bone tissue after severe injury has been a great challenge for regenerative medicine. Mounting evidence has shown that mesenchymal stem cells (MSCs) display great ex vivo expansion potential and differentiation properties, making them an attractive tool for bone tissue engineering. [1, 2] Achieving a better osteogenic differentiation efficacy is a central goal for MSCs-based bone regeneration field. The mechanism of MSCs lineage commitment is under control by multiple factors, including growth factors, transcription factors, and epigenetic factors. [3, 4] In particular, most of the epigenetic factors are enzymes, making them suitable targets for drug intervention. DNA methylation and histone modification are the major epigenetic regulation mechanisms. [5]

The activity of Runt-related transcription factor 2 (Runx2) [6], a master regulator of osteogenic differentiation, can be regulated by epigenetic factors. For example, the DNA methylation and acetylation of histones H3 and H4 status in the promoter of osteocalcin (OCN), which harbors binding sites for Runx2, can alter the accessibility of the promoter to Runx2. [7, 8] A study has shown that treatment of DNA-demethylating agent 5-aza-2′-deoxycytidine and chromatin-acetylation agent trichostatin A led to adipocyte differentiation in human-derived MSCs. [9]

Histone methylation plays a key role in establishing and maintaining stable gene expression patterns during cellular differentiation and embryonic development. [10] Histone demethylases KDM4B and KDM6B are essential in osteogenic commitment of MSCs via H3K9me3 and H3K27me3 modification, suggesting a promising strategy to improve osteogenic differentiation through manipulation of epigenetic factors. [3] However, the effect of inhibition of lysine-specific histone methyltransferases on MSCs differentiation has not been explored. Chaetocin is a fungal mycotoxin isolated from *Chaetomium minutum* and an inhibitor of lysine-specific histone methyltransferases, including SUV39H1 and G9a. [11, 12] It has been shown that inhibition H3K9me3-specific methyltransferase by Chaetocin can prevent cell growth in a ROS-dependent manner. [13] However, its potential influences on MSC differentiation are less known.

To evaluate the effect of Chaetocin on the differentiation of MSCs, we treated mouse-derived MSCs with Chaetocin and examined their osteogenic and adipogenic properties. We also checked the expression of osteogenic markers (Runx2 and OPN) and adipogenic markers (Pparg and Fabp4) during differentiation induction. Our results showed that Chaetocin promotes the osteogenic differentiation but inhibits the adipogenic differentiation of MSCs. In addition, treatment of Chaetocin in MSCs increases Wnt/*β*-catenin activity. Finally, we showed that bone formation of MSC in vivo is enhanced by treatment of Chaetocin. Our data uncovered a critical role of Chaetocin in MSC osteogenic differentiation and provide new insights into bone tissue engineering.

## 2. Materials and Methods

### 2.1. Ethics

All experimental protocols and procedures were approved by the Department of Stomatology, The Seventh People's Hospital of Shenzhen (protocol number 2018021001). The animal procedures were conducted in accordance with e-Guidelines for the Care and Use of Laboratory Animals of Department of Stomatology, The Seventh People's Hospital of Shenzhen.

### 2.2. MSC Isolation and Culture

MSCs were isolated from the femur and tibia bone marrow of adult c57bl/6 mice as described previously. [14] Cells were cultured in *α*-minimum essential medium (*α*-MEM; Thermo Fisher Scientific, China) supplemented with 10% fetal bovine serum (FBS; Gibco, China) and 100 *μ*g/ml penicillin-streptomycin (Gibco, China) in a humidified atmosphere containing 5% CO_2_ at 37°C. For Chaetocin experiment, 5 *μ*M Chaetocin (Selleck, China) was used. DMSO was used as control.

### 2.3. Osteoblast Differentiation and Analysis of MSCs

5 × 10^5^ MSCs were plated onto 6-well plates to reach ~80% confluence. The growth medium was then changed to osteoblast induction medium (OIM). The OIM was composed of high-glucose DMEM with L-glutamine (Thermo Fisher Scientific, China), 10% FBS (Gibco, China), 100 *μ*g/ml penicillin/streptomycin (Gibco, China), *β*-glycerophosphate 10 mM (Sigma Aldrich, China), 2.5 *μ*g/ml ascorbic acid-2-phosphate (Sigma Aldrich, China), 2.5 *μ*g/ml amphotericin B (Sigma Aldrich, China), and 0.1 *μ*M dexamethasone (Sigma Aldrich, China). For alkaline phosphatase (ALP) staining, MSCs cultured in OIM for 7 days were first fixed in 10% formalin for 1 h at room temperature and then stained by using an ALP staining kit (Sigma Aldrich, China) according to the manufacturer's instructions. ALP activity was assessed using the Alkaline Phosphatase Activity Detection Kit (Yeasen, China) according to the manufacturers protocol. For Alizarin Red S (ARS) staining, MSCs cultured in OIM for 14 days were stained 1% ARS solution for 20 minutes.

### 2.4. Adipogenic Differentiation and Analysis of MSCs

5 × 10^5^ MSCs were plated onto 6-well plates to reach ~80% confluence. The growth medium was then changed to adipogenic differentiation medium containing 10 *μ*g/ml insulin (Sigma Aldrich, China),1 *μ*M dexamethasone (Sigma Aldrich, China), and 500 *μ*M IBMX (Sigma Aldrich, China). Differentiated MSCs were first fixed with 10% formalin solution for 30 min and then stained with 0.3% oil red O solution for 10 min.

### 2.5. MSC-Mediated Ectopic Bone Formation

2 × 10^6^ MSCs were mixed with 40 *μ*g of tricalcium phosphate/hydroxyapatite (TCP/HA) powder (Sigma-Aldrich, China) and placed subcutaneously into nude mice (Kunming Model Animal Center, China). Mice were separated randomly into two groups of 8 animals each and were administered either with DMSO or Chaetocin (0.5 mg/kg body weight), intraperitoneally every other day. Tissues were then harvested 42 days for histological analysis. The tissue blocks were dehydrated and embedded in paraffin. The embedded tissue blocks were sliced in 5 *μ*m thickness and stained in hematoxylin and eosin staining solution. The sections were dehydrated, permeabilized, and sealed. The sections were observed under the microscope and photographed.

### 2.6. Real-Time Quantitative Reverse Transcription PCR (qRT-PCR)

Total RNA was extracted from cells using Trizol reagent (Invitrogen, China) according to the manufacturer's instructions. Reverse transcription was performed using 1 *μ*g of RNA using a MultiScribe reverse transcriptase kit (Applied Biosystems, China) according to the manufacturer's instructions. For qRT-PCR, a SYBR Green kit (Bio-Rad Laboratories Inc., China) was used according to the manufacturer's instructions. GAPDH was used as an internal control. The primer sequences for qRT-PCR are listed in Table 1.

### 2.7. Western Blot Analysis

Cells were lysed by using the Nuclear Extraction Kit (Novus Biologicals, China) to collect the nuclear protein. Samples were then used for electrophoresis separation, and then transferred to a nitrocellulose membrane. After blocked with 5% milk at room temperature for 2 hr, the membranes were added with primary antibodies to *β*-catenin (Abcam, China), Histone H1 (Abcam, China), Axin2 (Abcam, China), Myc (R&D Systems, China), Ccnd1 (Santa Cruz Biotechnology, USA) and GAPDH (Sigma, China) at 4°C overnight. After that, the membranes were incubated with secondary antibody (Abcam, China) at 37°C for 1 hr. The membranes were then completely immersed in the enhanced chemiluminescence (Yeasen, China) to obtain images.

### 2.8. Statistical Analysis

All data were presented as the mean ± s.d. Student's *t*-test was used between two groups and a difference was considered statistically significant with P <0.05. All statistical analyses were analyzed by using the SPSS 16.0 software.

## 3. Results

### 3.1. Chaetocin Promotes Osteogenic Differentiation of MSCs

**Figure 1:**
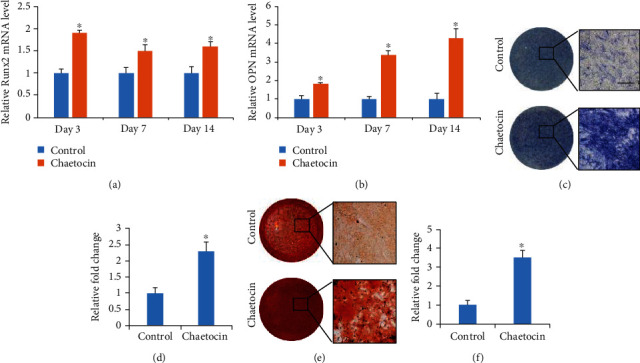
Effect of Chaetocin on MSC osteogenic differentiation. (a, b) Real-time RT-PCR analysis shows that expression of both early and later osteogenic markers (Runx2, OPN) was significantly enhanced by treatment with Chaetocin. (c, d) ALP staining showed a significant increase of ALP activity in MSCs treated with Chaetocin. (e, f) ARS staining showed a significant increase of calcium deposition in MSCs treated with Chaetocin. PCR and quantification data are expressed as means ± s.d. of three independent experiments (∗*p* < 0.05). Scale bar: 100 *μ*m.

The status histone methylation plays a crucial role in regulating chromatin structural changes and determines the accessibility of related gene promoters for transcription factors during MSC differentiation. To determine the effects of the histone methyltransferase inhibitor Chaetocin on osteogenic differentiation, we first examined the expression of the osteogenesis-related genes in MSCs during osteogenic induction (Figures 1(a) and 1(b)). Compared to the control MSCs, the mRNA expression of *Runx2* and *OPN* were upregulated in the cells treated with Chaetocin at day 3, 7 and 14 (Figures 1(a) and 1(b)). We further explored effects of Chaetocin on the osteogenic differentiation by performing the ALP staining and ARS staining to detect ALP activity and mineralization (Figures 1(c) and 1(d)). Compared to the control, ALP activity and mineralization were significantly increased in the MSCs treated with Chaetocin (Figures 1(c) and 1(d)). Thus, treatment of Chaetocin could promote osteogenic differentiation of MSCs.

### 3.2. Chaetocin Inhibits Adipogenic Differentiation of MSCs

**Figure 2:**
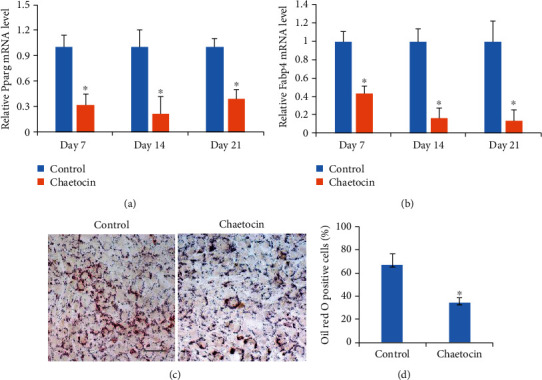
Effect of Chaetocin on MSC adipogenic differentiation. (a, b) Real-time RT-PCR analysis shows that expression adipogenic markers (Pparg, Fabp4) was significantly inhibited by treatment with Chaetocin. (c, d) Oil red O staining showed a significant decrease of oil droplets in MSCs treated with Chaetocin. PCR and quantification data are as expressed as means ± s.d. of three independent experiments (∗*p* < 0.05). Scale bar: 500 *μ*m.

Previous studies have shown that MSCs are associated with multiple lineages, including osteoblast, adipocytes, chondrocytes, and so on. The association of MSCs with adipocytes resulted in the imbalance between bone mass and fat and the increased risk of bone fracture. Hence, we sought to investigate the effects of Chaetocin on adipogenic differentiation of MSCs. Compared to the control MSCs, the mRNA expression of adipogenesis-related genes, *Pparg* and *Fabp4*, was dramatically reduced in the MSCs treated with Chaetocin (Figures 2(a) and 2(b)). Further, oil red O staining results showed that oil droplet formation was significantly inhibited in the MSCs treated with Chaetocin (Figures 2(c) and 2(d)). Thus, treatment of Chaetocin could inhibit adipogenesis of MSCs.

### 3.3. Chaetocin Activates Wnt/*β*-Catenin Activity during Osteogenic Induction

**Figure 3:**
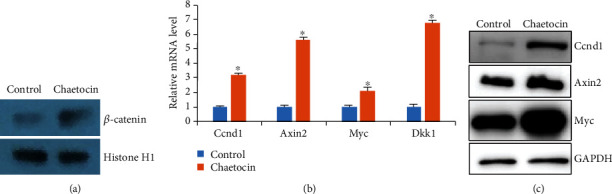
Treatment of Chaetocin increase Wnt/*β*-catenin activities. (a) Western blot shows nuclear *β*-catenin protein levels were elevated by Chaetocin treatment in MSC culture at day 7. Histone H1 was used as a loading control. (b) Real-time RT-PCR analysis shows that Wnt/*β*-catenin target genes (Cnnd1, Axin2, Myc, and Dkk1) were significantly increased by treatment with Chaetocin. PCR data is expressed as means ± s.d. of three independent experiments (∗*p* < 0.05). (c) WB results show that Cnnd1, Axin2, and Myc were significantly increased after Chaetocin treatment.

As we showed that Chaetocin inhibited adipogenic genes while promoted osteogenic gene expression. It seems that Chaetocin may not necessarily direct regulate the H3K9me3 levels of these particular genes. Since Wnt/*β*-catenin activity has been well-known for its function in promote osteogenesis and inhibit the adipogenesis of MSCs, we reasoned that Chaetocin might regulates Wnt/*β*-catenin signaling transduction in MSCs. Indeed, our Western Blot results showed that the level of nuclear *β*-catenin in MSCs treated with Chaetocin was higher compared with control MSCs (Figure 3(a)). The mRNA expression levels of Wnt/*β*-catenin target genes (*Ccnd1*, *Axin2*, *Myc*, and *Dkk1*) were also increased in MSCs treated with Chaetocin (Figure 3(b)), indicating that Chaetocin is involved in Wnt/*β*-catenin signaling transduction.

### 3.4. Chaetocin Promotes Ectopic Osteogenesis In Vivo

**Figure 4:**
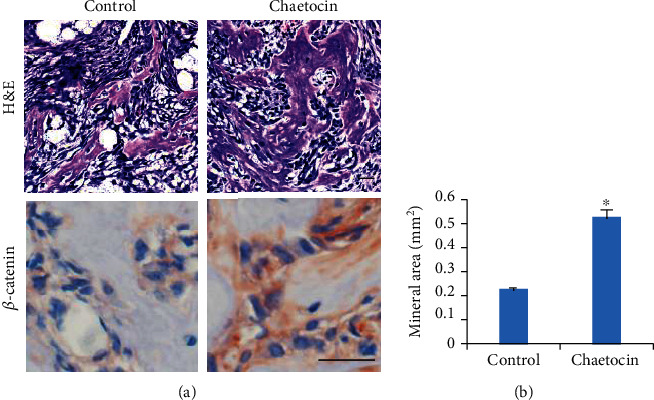
Chaetocin promotes MSC-mediated ectopic bone formation. (a) H&E staining results show more bone tissues from MSCs treated with Chaetocin compared with control. IHC results showed more nuclear *β*-catenin signal in MSC treated with Chaetocin compared with control. Scale bar: 50 *μ*m. (b) Quantification of the bone area from MSCs treated with Chaetocin and control. Quantification data is expressed as means ± s.d. of three independent experiments (∗*p* < 0.05).

To determine whether Chaetocin could play a role in MSC-mediated bone formation in vivo, MSCs were mixed with TCP/HA and injected into nude mice. Mice were then treated with control vehicle or Chaetocin for 6 weeks and sacrificed for sample collection. HE staining (Figure 4(a)) showed both the control group and the experiment group were found to show osteoblast-like cells. Quantification of the bone area showed that treatment of Chaetocin led to the increase of bone tissue in vivo (Figure 4(b)). In addition, our immunohistochemistry staining of *β*-catenin results confirm the presence of nuclear *β*-catenin signal in the sample treated with Chaetocin. Overall, our data demonstrated that Chaetocin could enhance MSC-based bone formation.

## 4. Discussion

MSCs have multiple differentiation potentials and can be induced to differentiate into two mutually exclusive lineages: osteoblasts or adipocytes. [15] How to induce MSCs into osteoblasts more efficiently has been a major challenge in bone tissue engineering. Previous studies have shown that histone methylation is involved in MSC differentiation. For example, KDM4B and KDM6B played a critical role in MSC cell fate commitment by removing H3K9me3 and H3K27me3 on different sets of lineage-specific genes. [3] In addition, H3K27 methyltransferase EZH2 is required for inhibition of MSC differentiation. [16] Chaetocin was originally identified as an inhibitor of histone methyltransferase SU(VAR)3-9. [11] The role of Chaetocin in anticancer treatment has been intensively studied; however, its function in MSCs differentiation has not been explored.

In this study, we showed that Chaetocin can affect the osteogenic and adipogenic abilities of MSCs. Treatment of Chaetocin promoted the osteogenic differentiation of MSCs and induced the expression of osteogenic-related genes. On the contrary, Chaetocin repressed the adipogenic differentiation of MSCs and reduced the number of oil droplets. Chaetocin can inhibit the activity of histone methyltransferase SUV39 family, such as SUV39H1 and G9a, which are required for H3K9 di- to tri-methylation and mono- to dimethylation of H3K9, respectively. [17, 18] Although the specific molecular mechanism by which Chaetocin might affect MSC osteogenesis and adipogenesis is not characterized in the current study, it is highly possible that MSC differentiation regulated by Chaetocin is mediated via alteration of the status of H3K9 methylation. In addition, how Chaetocin can exert a different function in a different context is also elusive, we speculate that Chaetocin can affect the osteo- and adipolineage commitment through Wnt/*β*-catenin activity instead of regulation of these lineage factors directly.

Our data also showed that treatment of Chaetocin in MSCs promoted Wnt/*β*-catenin activity. Interestingly, several histone demethylases have been linked to Wnt/*β*-catenin activity. For instance, KDM7A can regulate adipogenic and osteogenic differentiation via regulation of Wnt/*β*-catenin signaling. [19] Furthermore, JMJD2D can interact with *β*-catenin to activate transcription of its target genes. [20] Hence, it is possible that Chaetocin-mediated inhibition of histone methyltransferase is involved in Wnt/*β*-catenin signaling. And indeed, both our cell culture assay and in vivo bone formation experiment showed that Wnt/*β*-catenin is activated when treated with Chaetocin. There is speculation if Chaetocin could be used in patient with osteoporosis to improve the formation of bone mass.

In conclusion, we demonstrate that treatment with Chaetocin can improve the osteogenesis of MSCs via epigenetic regulation. Our study provides useful insights for better exploring the use of Chaetocin in bone tissue engineering.

## Figures and Tables

**Table 1:** Primer information for qRT-PCR.

Primer name	Primer sequence
Runx2 forward	5′-GACTGTGGTTACCGTCATGGC-3′
Runx2 reverse	5′-ACTTGGTTTTTCATAACAGCGGA-3′
OPN forward	5′-CTGCATACTGTAACCGCAGTC-3′
OPN reverse	5′-CTCTCCATCCATAACATGGGC-3′
Pparg forward	5′-TCCTGTAAAAGCCCGGAGTAT-3′
Pparg reverse	5′-GCTCTGGTAGGGGCAGTGA-3′
Fabp4 forward	5′-AAGGTGAAGAGCATCATAACCCT-3′
Pparg reverse	5′-TCACGCCTTTCATAACACATTCC-3′
Ccnd1 forward	5′-GCGTACCCTGACACCAATCTC-3′
Ccnd1 reverse	5′-CTCCTCTTCGCACTTCTGCTC-3′
Myc forward	5′-TTCTACGACTATGACTGCGGA-3′
Myc reverse	5′-TGATGGAAGCATAATTCCTGCC-3′
Axin2 forward	5′-TGACTCTCCTTCCAGATCCCA-3′
Axin2 reverse	5′-TGCCCACACTAGGCTGACA-3′
Dkk1 forward	5′-CTCATCAATTCCAACGCGATCA-3′
Dkk1 reverse	5′-GCCCTCATAGAGAACTCCCG-3′
GAPDH forward	5′-TGGATTTGGACGCATTGGTC-3′
GAPDH reverse	5′-TTTGCACTGGTACGTGTTGAT-3′

## Data Availability

The data used to support the findings of this study are included within the article.

## References

[1] Oryan A., Kamali A., Moshiri A., Baghaban E. M. (2017). Role of Mesenchymal stem cells in bone regenerative medicine: what is the evidence?. *Cells, Tissues, Organs*.

[2] Zhao C., Zeng Z., Qazvini N. T. (2018). Thermoresponsive Citrate-Based Graphene Oxide Scaffold Enhances Bone Regeneration from BMP9-Stimulated Adipose-Derived Mesenchymal Stem Cells. *ACS Biomaterials Science & Engineering*.

[3] Ye L., Fan Z., Yu B. (2012). Histone demethylases KDM4B and KDM6B promotes osteogenic differentiation of human MSCs. *Cell Stem Cell*.

[4] Almalki S. G., Agrawal D. K. (2016). Key transcription factors in the differentiation of mesenchymal stem cells. *Differentiation*.

[5] Teven C. M., Liu X., Hu N. (2011). Epigenetic regulation of mesenchymal stem cells: a focus on osteogenic and adipogenic differentiation. *Stem Cells International*.

[6] Komori T. (2017). Roles of Runx2 in skeletal development. *Advances in Experimental Medicine and Biology*.

[7] Villagra A., Gutiérrez J., Paredes R. (2002). Reduced CpG methylation is associated with transcriptional activation of the bone-specific rat osteocalcin gene in osteoblasts. *Journal of Cellular Biochemistry*.

[8] Shen J., Hovhannisyan H., Lian J. B. (2003). Transcriptional induction of the osteocalcin gene during osteoblast differentiation involves acetylation of histones h3 and h4. *Molecular Endocrinology*.

[9] Zych J., Stimamiglio M. A., Senegaglia A. C. (2013). The epigenetic modifiers 5-aza-2'-deoxycytidine and trichostatin A influence adipocyte differentiation in human mesenchymal stem cells. *Brazilian Journal of Medical and Biological Research*.

[10] Yang D., Yu B., Sun H., Qiu L. (2017). The Roles of Histone Demethylase Jmjd3 in Osteoblast Differentiation and Apoptosis. *Journal of Clinical Medicine*.

[11] Greiner D., Bonaldi T., Eskeland R., Roemer E., Imhof A. (2005). Identification of a specific inhibitor of the histone methyltransferase SU(VAR)3-9. *Nature Chemical Biology*.

[12] Iwasa E., Hamashima Y., Fujishiro S. (2010). Total synthesis of (+)-chaetocin and its analogues: their histone methyltransferase G9a inhibitory activity. *Journal of the American Chemical Society*.

[13] Chaib H., Nebbioso A., Prebet T. (2012). Anti-leukemia activity of chaetocin via death receptor-dependent apoptosis and dual modulation of the histone methyl-transferase SUV39H1. *Leukemia*.

[14] Soleimani M., Nadri S. (2009). A protocol for isolation and culture of mesenchymal stem cells from mouse bone marrow. *Nature Protocols*.

[15] Barui A., Chowdhury F., Pandit A., Datta P. (2018). Rerouting mesenchymal stem cell trajectory towards epithelial lineage by engineering cellular niche. *Biomaterials*.

[16] Wei Y., Chen Y.-H., Li L.-Y. (2011). CDK1-dependent phosphorylation of EZH2 suppresses methylation of H3K27 and promotes osteogenic differentiation of human mesenchymal stem cells. *Nature Cell Biology*.

[17] Rice J. C., Briggs S. D., Ueberheide B. (2003). Histone methyltransferases direct different degrees of methylation to define distinct chromatin domains. *Molecular Cell*.

[18] Peters A. H. F. M., Kubicek S., Mechtler K. (2003). Partitioning and plasticity of repressive histone methylation states in mammalian chromatin. *Molecular Cell*.

[19] Yang X., Wang G., Wang Y. (2019). Histone demethylase KDM7A reciprocally regulates adipogenic and osteogenic differentiation via regulation of C/EBP*α* and canonical Wnt signalling. *Journal of Cellular and Molecular Medicine*.

[20] Peng K., Kou L., Yu L. (2019). Histone demethylase JMJD2D interacts with *β*-catenin to induce transcription and activate colorectal cancer cell proliferation and tumor growth in mice. *Gastroenterology*.

